# Gene Expression Profiles of the Aging Rat Hippocampus Imply Altered Immunoglobulin Dynamics

**DOI:** 10.3389/fnins.2022.915907

**Published:** 2022-05-25

**Authors:** Panagiotis Giannos, Konstantinos Prokopidis

**Affiliations:** ^1^Department of Life Sciences, Faculty of Natural Sciences, Imperial College London, London, United Kingdom; ^2^Society of Meta-Research and Biomedical Innovation, London, United Kingdom; ^3^Department of Musculoskeletal Biology, Institute of Life Course and Medical Sciences, University of Liverpool, Liverpool, United Kingdom

**Keywords:** hippocampus, aging, rat brain, differentially expressed genes, gene expression, immunoglobulins, blood-brain barrier

## Abstract

Aging is a process that leads to the deterioration in physiological functioning of the brain. Prior research has proposed that hippocampal aging is accompanied by genetic alterations in neural, synaptic, and immune functions. Nevertheless, interactome-based interrogations of gene alterations in hippocampal aging, remain scarce. Our study integrated gene expression profiles of the hippocampus from young and aged rats and functionally classified network-mapped genes based on their interactome. Hippocampal differentially expressed genes (DEGs) between young (5–8 months) and aged (21–26 months) male rats (*Rattus norvegicus*) were retrieved from five publicly available datasets (GSE14505, GSE20219, GSE14723, GSE14724, and GSE14725; 38 young and 29 aged samples). Encoded hippocampal proteins of age-related DEGs and their interactome were predicted. Clustered network DEGs were identified and the highest-ranked was functionally annotated. A single cluster of 19 age-related hippocampal DEGs was revealed, which was linked with immune response (biological process, *P* = 1.71E-17), immunoglobulin G binding (molecular function, *P* = 1.92E-08), and intrinsic component of plasma membrane (cellular component, *P* = 1.25E-06). Our findings revealed dysregulated hippocampal immunoglobulin dynamics in the aging rat brain. Whether a consequence of neurovascular perturbations and dysregulated blood-brain barrier permeability, the role of hippocampal immunoregulation in the pathobiology of aging warrants further investigation.

## Introduction

Aging is a ubiquitous yet inevitable biological phenomenon that drives the transient deterioration in physiological functioning of the brain. Brain aging is associated with cognitive decline including memory and executive function, perturbations which often correlate with age-driven structural alterations that are accompanied by neuronal loss and synaptic dysfunction (López-Otín et al., 2013). Aging also constitutes the primary risk factor for many neurodegenerative diseases, including Alzheimer’s and Parkinson’s disease, of which prevalence increases with advancing age (Kennedy et al., 2014).

The hippocampus is a brain locus fundamental to cognition, with a profound role in learning and memory consolidation (Jack et al., 1999). Prior research has documented that structural and functional changes in the hippocampus correlate with development and disease severity of neurodegenerative disorders linked with cognitive decline. Previous studies have revealed that hippocampal aging is similar in humans and in animal models, with prominent genetic dysregulation in neural, synaptic and immune functions.

Neurobiological alterations seen in the aging brain can precede apparent histopathological degeneration of the brain, suggesting that analysis of brain gene expression may offer unique insights into the molecular mechanisms underlying age-related changes (Lu et al., 2004). At present, interactome mapping and network-based interrogations of gene-specific targets bearing functional significance in hippocampal aging, remain largely unexplored. In this study, we integrated publicly available gene expression profiles of the hippocampus from young and aged rats to identify putative alterations in hippocampal processes underlying aging.

## Materials and Methods

### Collection of Microarray Data

We screened the literature through the Gene Expression Omnibus for relevant peer-reviewed datasets based on organism type (*Rattus norvegicus*), expression profiling (microarray), sample type (brain hippocampal tissue), and condition (aging), using the search terms “aged” or “aging” or “old” or “young” and “brain” or “hippocampus.” No restrictions based on language and geographic origin were used in our search, while no exclusion criteria in the baseline characteristics of animals from which tissue sections were obtained, were applied. Duplicate gene expression sample and series or those lacking expression data for controls or with incompatible platforms, were excluded. Author (PG) formulated the search strategy and in conjunction with a second author (KP), the yielded datasets were screened and any discrepancies in the literature search were resolved.

### Identification of Differentially Expressed Genes

Brain hippocampal samples from healthy young and aged rat models were compared and differentially expressed genes (DEGs) were retrieved using ImaGEO (Toro-Domínguez et al., 2019). Significance analysis and integration were conducted using the fixed effect model to identify those DEGs with the strongest average effect across the collected datasets. This approach was followed as both the treatment (i.e., aging) and expression array platforms were orthogonal between datasets. Genes with a *P* < 0.05 corrected by the Benjamini-Hochberg (BH) false discovery rate (FDR) were regarded as significant. DEGs with *Z* score > 1.96 were classified as upregulated, while those with *Z* score < 1.96 as downregulated (corresponding to a significance level of 5%).

### Prediction of Network-Based Protein Interactions

Encoded hippocampal proteins of the aging DEGs and their interactome were predicted into a protein-protein interaction (PPI) network using The Search Tool for the Retrieval of Interacting Genes *via* a medium probabilistic confidence score of >0.4 and mapped with Cytoscape (Szklarczyk et al., 2019). A reasonably moderate cut-off score was ensued to amplify the coverage of all potential protein interactions without inflating their precision. Proteins lacking any interactions were excluded from the network.

### Identification of Clustering Modules and Characterization of Gene Hubs

Central gene elements of the aging rat hippocampal network were inferred by measuring network features from their complex interactome and by identifying sub-networks and hub objects (Giannos et al., 2021a,b, 2022). Highly clustered DEGs or densely connected modules in the PPI network were identified using the molecular complex detection (Bader and Hogue, 2003). Cut-off selection was ensued based on the default network scoring parameters: degree cut-off = 2, haircut cluster finding, node score cut-off = 0.2, *K*-core = 2, and max depth = 100. The interactome interference of module DEGs in the PPI network was quantified using CytoHubba (Chin et al., 2014). Module DEGs were ranked based on the intersection of 11 established topological algorithms, as previously described: Degree, Closeness, Betweenness, Radiality, Stress, EcCentricity, BottleNeck, Edge Percolated Component, Maximum Neighborhood Component, Density of Maximum Neighborhood Component, and Maximal Clique Centrality.

### Functional Annotation Enrichment Analysis

Enrichment of aging DEGs in the rat hippocampus was predicted using Gene Ontology (GO) annotations from the Molecular Signatures Database (Liberzon et al., 2015). Functional classification was ensued using a probability density *P* < 0.05 following BH FDR correction and categorized into three groups of GO terms: biological process (BP), molecular function (MF), and cellular component (CC).

## Results

### Overview of Microarray Datasets

**FIGURE 1:**
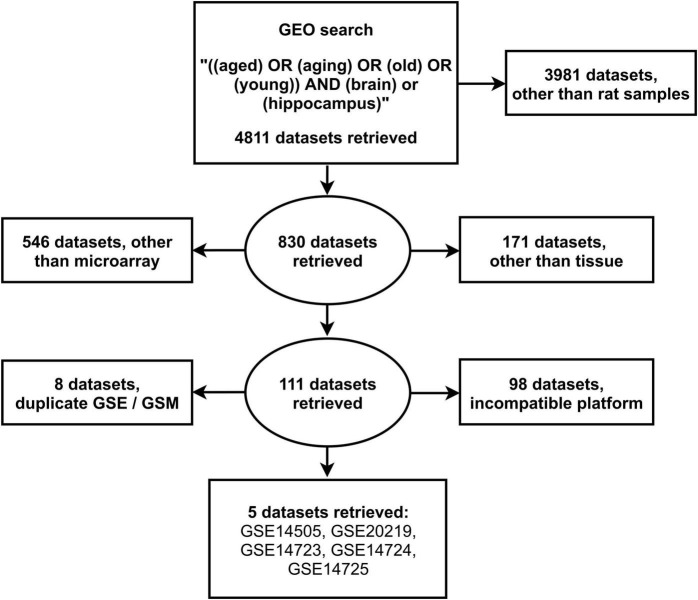
Search strategy for the selection of eligible gene expression datasets from the National Center for Biotechnology Information Gene Expression Omnibus.

The search of the GEO database resulted in 4811 datasets, of which 3981 contained gene expression data from organisms other than rats. From these, 111 microarray datasets were retrieved after exclusion of 717 datasets which included non-profiling by array expression data from samples other than tissues. Further exclusion of 106 studies with either duplicate gene expression samples or series and incompatible gene expression platform resulted in 5 eligible datasets: GSE14505 (Nagahara et al., 2009), GSE20219 (Blalock et al., 2010), GSE14723 (Haberman et al., 2011), GSE14724 (Haberman et al., 2011), and GSE14725 (Haberman et al., 2011; Figure 1). The retrieved datasets consisted of 38 young (5–8 months) and 29 aged (21–26 months) male rat hippocampal tissue samples (Supplementary Table 1).

### Differentially Expressed Genes in Hippocampal Aging

Integration analysis revealed a total of 527 aging DEGs, of which 278 showed significantly increased hippocampal expression while 249 DEGs decreased expression in the rat brain (Supplementary Table 2).

### Protein-Protein Interaction Network and Functional Modules in Hippocampal Aging

**FIGURE 2:**
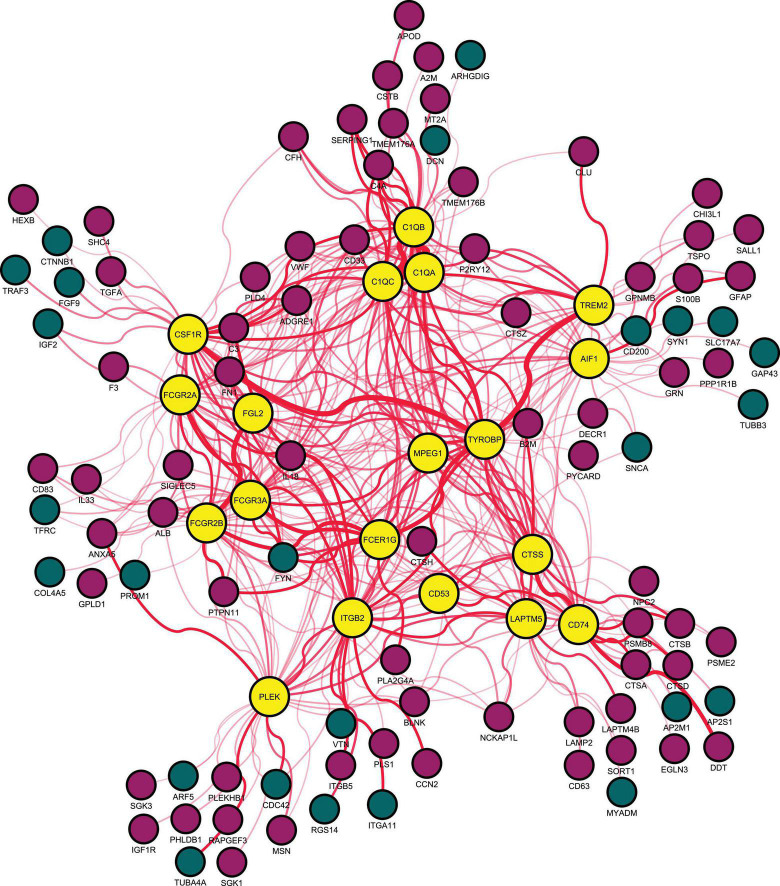
Highest-ranked clustering gene module in the protein-protein interaction network of differentially expressed genes between young (5–8 months) and aged (21–26 months) of the rat hippocampus. Red indicates upregulated and blue downregulated node genes. AIF1, allograft inflammatory factor 1; C1QA, complement C1q A chain; C1QB, complement C1q B chain; C1QC, complement C1q C chain; CD53, CD53 molecule; CD74, CD74 molecule; CSF1R, colony stimulating factor 1 receptor; CTSS, cathepsin S; FCER1G, Fc epsilon receptor Ig; FCGR2A, Fc gamma receptor IIa; FCGR2B, Fc gamma receptor IIb; FCGR3A, Fc gamma receptor IIIa; FGL2, fibrinogen like 2; ITGB2, integrin subunit beta 2; LAPTM5, lysosomal protein transmembrane 5; MPEG1, macrophage expressed 1; PLEK, pleckstrin; TREM2, triggering receptor expressed on myeloid cells 2; and TYROBP, transmembrane immune signaling adaptor TYROBP.

A PPI network of DEGs in the aging rat hippocampus was constructed and consisted of a total of 474 encoded proteins with 1,862 interactions. A single highest-ranked clustering module was retrieved and consisted of 19 upregulated aging DEGs with 362 interactions among 92 genes: allograft inflammatory factor 1 (AIF1), complement C1q A chain (C1QA), complement C1q B chain (C1QB), complement C1q C chain (C1QC), CD53 molecule (CD53), CD74 molecule (CD74), colony stimulating factor 1 receptor (CSF1R), cathepsin S (CTSS), Fc epsilon receptor Ig (FCER1G), Fc gamma receptor IIa (FCGR2A), Fc gamma receptor IIb (FCGR2B), Fc gamma receptor IIIa (FCGR3A), fibrinogen like 2 (FGL2), integrin subunit beta 2 (ITGB2), lysosomal protein transmembrane 5 (LAPTM5), macrophage expressed 1 (MPEG1), pleckstrin (PLEK), triggering receptor expressed on myeloid cells 2 (TREM2), and transmembrane immune signaling adaptor TYROBP (TYROBP; Figure 2, Table 1, and Supplementary Table 3). Multi-algorithmic topological analysis revealed MPEG1 as the highest ranked hub gene (Supplementary Table 4). DEGs in the highest-ranked clustering module were most enriched with immune response (BP, *P* = 1.71E-17), immunoglobulin G binding (MF, *P* = 1.92E-08), and intrinsic component of plasma membrane (CC, *P* = 1.25E-06; Supplementary Table 5).

**TABLE 1:** Characteristics of the highest-ranked clustering gene module in the protein-protein interaction network of differentially expressed genes of the hippocampus between young (5–8 months) and aged (21–26 months) rats.

Gene ID	*P*-value	*Z*-score	Gene name
AIF1	1.32E-02	3.64	Allograft inflammatory factor 1
C1QA	3.47E-09	7.00	Complement C1q A chain
C1QB	5.43E-04	4.60	Complement C1q B chain
C1QC	2.90E-06	5.79	Complement C1q C chain
CD53	3.98E-07	6.15	CD53 molecule
CD74	1.36E-08	6.75	CD74 molecule
CSF1R	4.79E-03	3.98	Colony stimulating factor 1 receptor
CTSS	8.61E-09	6.83	Cathepsin S
FCER1G	4.80E-10	7.34	Fc epsilon receptor Ig
FCGR2A	2.85E-02	3.35	Fc gamma receptor IIa
FCGR2B	4.78E-09	6.93	Fc gamma receptor IIb
FCGR3A	3.36E-02	3.29	Fc gamma receptor IIIa
FGL2	1.25E-05	5.46	Fibrinogen like 2
ITGB2	2.48E-03	4.18	Integrin subunit beta 2
LAPTM5	3.70E-06	5.73	Lysosomal protein transmembrane 5
MPEG1	5.75E-04	4.58	Macrophage expressed 1
PLEK	2.67E-03	4.16	Pleckstrin
TREM2	7.34E-04	4.52	Triggering receptor expressed on myeloid cells 2
TYROBP	9.92E-09	6.80	Transmembrane immune signaling adaptor TYROBP

## Discussion

Analysis of DEGs from hippocampal tissues from young (5–8 months) and aged (21–26 months) rats, identified a single highly clustered gene module consisted of 19 upregulated DEGs: AIF1, C1QA, C1QB, C1QC, CD53, CD74, CSF1R, CTSS, FCER1G, FCGR2A, FCGR2B, FCGR3A, FGL2, ITGB2, LAPTM5, MPEG1, PLEK, TREM2, and TYROBP. Functional annotations of these DEGs revealed an association with ontology processes of immune response and immunoglobulin G binding, and properties of an intrinsic component of plasma membrane. Our findings revealed dysregulated hippocampal immunoglobulin dynamics both in terms of expression and interactions in the aging rat brain, rendering these DEGs as a potential marker of putative neurogenetic alterations during aging.

Dysfunctions of the blood brain barrier (BBB) are often linked with increased peripheral uptake of immunoglobulins and other plasma proteins in the brain (Bake et al., 2009). Post-mortem insights have demonstrated that breakdown of BBB in response to neurodegeneration is characterized by accumulation in hippocampal blood-derived immunoglobulins. Dysregulated BBB permeability is characterized by perturbations in the crosstalk among brain endothelial cells, pericytes, and astrocytes of the neurovascular microenvironment (Montagne et al., 2015). Aging related neurovascular unit dysfunction may arise from normal cell senescence or pathologically under age-related distress, such as cerebrovascular accident or reperfusion injury (i.e., ischemic stroke) and predisposition to neurogenetic-driven degeneration (i.e., Parkinson’s disease and Alzheimer’s disease), with peripheral immune penetration leading disease progression (Cai et al., 2017; Li et al., 2019).

Genomic stability is an essential requirement for the maintenance of functional and structural integrity of the BBB. Accumulation of DNA damage in terms of DNA breaks, cross-links, and bases mismatches (McKinnon, 2013; Lodato et al., 2018) and aberrations across the DNA repair machinery (Santos et al., 2013; Chow and Herrup, 2015; Zhao et al., 2017), have all been described during aging (Li et al., 2019). In this way, altered DNA damage responses and repair signaling likely underlie the penetration of blood-derived substances and the dysregulation of immunity in the aging hippocampus, both of which are intimately linked with neurodegenerative progression (Lucin and Wyss-Coray, 2009). Whether a consequence of neurovascular dysfunction and dysregulated blood-brain barrier permeability, the role of hippocampal immunoregulation in the pathobiology of aging warrants further investigation.

### Strengths and Limitations

This is the first study that comprehensively examined the potential role of DEGs and their interactome as gene biomarkers in hippocampal aging using 5 publicly available datasets with almost 70 included rat samples and using a multi-algorithmic protein-interaction based functional approach that was dependent on different levels of annotation. Nevertheless, certain conceptual and methodological limitations exist. Hippocampal tissue distribution from which gene expression was derived in the included studies was sparsely heterogeneous, ranging from whole hippocampus samples to those from dorsal and ventral areas, the CA1/CA3 regions or the dentate gyrus. Although histologically distinguishable subfields, these likely share common genomic organizations and thus, aging related accumulation of DNA damage from these subregions may be overlapping (Van der Meer et al., 2020). Moreover, despite that the expression profiling platforms employed by the included datasets were indifferent, lab effects due to experimental variation are known to hinder statistical power in DEGs detection, a challenge which remains even after normalization (Johnson et al., 2007; Roberts et al., 2011; Hansen et al., 2012; Dillies et al., 2013; Lyu and Li, 2016). Yet, an attempt to address unknown cross-study heterogeneity was made by integrating gene expression rather than considering their overlap between datasets.

## Conclusion

Immunoglobulin dysregulation distinguishes hippocampal gene expression between aged and young rat brains. Increased awareness of the potential role of hippocampal-focused immunity in the pathobiology of aging, may reveal whether immunoglobulin dysregulation forms the culprit of hippocampal aging and not merely an epiphenomenon of aging-related alterations in BBB.

## Data Availability Statement

Publicly available datasets were analyzed in this study. These are available in the Gene Expression Omnibus repository, under the following accession numbers: GSE14505, GSE20219, GSE14723, GSE14724, and GSE1472.

## Author Contributions

PG conceived and designed the study. PG and KP acquired, collated, analyzed the data, and drafted and revised the manuscript critically for important intellectual content. Both authors gave final approval of the version to be published and have contributed to the manuscript.

## Conflict of Interest

The authors declare that the research was conducted in the absence of any commercial or financial relationships that could be construed as a potential conflict of interest.

## Publisher’s Note

All claims expressed in this article are solely those of the authors and do not necessarily represent those of their affiliated organizations, or those of the publisher, the editors and the reviewers. Any product that may be evaluated in this article, or claim that may be made by its manufacturer, is not guaranteed or endorsed by the publisher.

## References

[B1] Bader G. D. Hogue C. W. (2003). An automated method for finding molecular complexes in large protein interaction networks. *BMC Bioinform.* 4:2. 10.1186/1471-2105-4-2 PMC149346 12525261

[B2] Bake S. Friedman J. A. Sohrabji F. (2009). Reproductive age-related changes in the blood brain barrier: expression of IgG and tight junction proteins. *Microvasc. Res.* 78 413–424. 10.1016/j.mvr.2009.06.009 19591848 PMC2784250

[B3] Blalock E. M. Phelps J. T. Pancani T. Searcy J. L. Anderson K. L. Gant J. C. (2010). Effects of long-term pioglitazone treatment on peripheral and central markers of aging. *PLoS One* 5:e10405. 10.1371/journal.pone.0010405 20454453 PMC2861595

[B4] Cai W. Zhang K. Li P. Zhu L. Xu J. Yang B. (2017). Dysfunction of the neurovascular unit in ischemic stroke and neurodegenerative diseases: an aging effect. *Ageing Res. Rev.* 34 77–87. 10.1016/j.arr.2016.09.006 27697546 PMC5384332

[B5] Chin C.-H. Chen S.-H. Wu H.-H. Ho C.-W. Ko M.-T. Lin C.-Y. (2014). cytoHubba: identifying hub objects and sub-networks from complex interactome. *BMC Syst. Biol.* 8:S11. 10.1186/1752-0509-8-S4-S11 25521941 PMC4290687

[B6] Chow H. M. Herrup K. (2015). Genomic integrity and the ageing brain. *Nat. Rev. Neurosci.* 16 672–684. 10.1038/nrn4020 26462757

[B7] Dillies M.-A. Rau A. Aubert J. Hennequet-Antier C. Jeanmougin M. Servant N. (2013). A comprehensive evaluation of normalization methods for Illumina high-throughput RNA sequencing data analysis. *Brief. Bioinform.* 14 671–683. 10.1093/bib/bbs046 22988256

[B8] Giannos P. Kechagias K. S. Bowden S. Tabassum N. Paraskevaidi M. Kyrgiou M. (2021a). PCNA in Cervical Intraepithelial Neoplasia and Cervical Cancer: an Interaction Network Analysis of Differentially Expressed Genes. *Front. Oncol.* 11:779042. 10.3389/fonc.2021.779042 34900731 PMC8661029

[B9] Giannos P. Kechagias K. S. Gal A. (2021b). Identification of Prognostic Gene Biomarkers in Non-Small Cell Lung Cancer Progression by Integrated Bioinformatics Analysis. *Biology* 10:1200. 10.3390/biology10111200 34827193 PMC8615219

[B10] Giannos P. Triantafyllidis K. K. Giannos G. Kechagias K. S. (2022). SPP1 in infliximab resistant ulcerative colitis and associated colorectal cancer: an analysis of differentially expressed genes. *Eur. J. Gastroenterol. Hepatol.* 34 598–606. 10.1097/MEG.0000000000002349 35102110

[B11] Haberman R. P. Colantuoni C. Stocker A. M. Schmidt A. C. Pedersen J. T. Gallagher M. (2011). Prominent hippocampal CA3 gene expression profile in neurocognitive aging. *Neurobiol. Aging* 32 1678–1692. 10.1016/j.neurobiolaging.2009.10.005 19913943 PMC2891610

[B12] Hansen K. D. Irizarry R. A. Wu Z. (2012). Removing technical variability in RNA-seq data using conditional quantile normalization. *Biostatistics* 13 204–216. 10.1093/biostatistics/kxr054 22285995 PMC3297825

[B13] Jack C. R. Petersen R. C. Xu Y. C. O’Brien P. C. Smith G. E. Ivnik R. J. (1999). Prediction of AD with MRI-based hippocampal volume in mild cognitive impairment. *Neurology* 52 1397–1397. 10.1212/wnl.52.7.1397 10227624 PMC2730146

[B14] Johnson W. E. Li C. Rabinovic A. (2007). Adjusting batch effects in microarray expression data using empirical Bayes methods. *Biostatistics* 8 118–127. 10.1093/biostatistics/kxj037 16632515

[B15] Kennedy B. K. Berger S. L. Brunet A. Campisi J. Cuervo A. M. Epel E. S. (2014). Geroscience: linking aging to chronic disease. *Cell* 159 709–713. 10.1016/j.cell.2014.10.039 25417146 PMC4852871

[B16] Li Y. Xie L. Huang T. Zhang Y. Zhou J. Qi B. (2019). Aging neurovascular unit and potential role of DNA damage and repair in combating vascular and neurodegenerative disorders. *Front. Neurosci.* 13:778. 10.3389/fnins.2019.00778 31440124 PMC6694749

[B17] Liberzon A. Birger C. Thorvaldsdóttir H. Ghandi M. Mesirov J. P. Tamayo P. (2015). The molecular signatures database hallmark gene set collection. *Cell Syst.* 1 417–425. 10.1016/j.cels.2015.12.004 26771021 PMC4707969

[B18] Lodato M. A. Rodin R. E. Bohrson C. L. Coulter M. E. Barton A. R. Kwon M. (2018). Aging and neurodegeneration are associated with increased mutations in single human neurons. *Science* 359 555–559. 10.1126/science.aao4426 29217584 PMC5831169

[B19] López-Otín C. Blasco M. A. Partridge L. Serrano M. Kroemer G. (2013). The hallmarks of aging. *Cell* 153 1194–1217. 23746838 10.1016/j.cell.2013.05.039 PMC3836174

[B20] Lu T. Pan Y. Kao S.-Y. Li C. Kohane I. Chan J. (2004). Gene regulation and DNA damage in the ageing human brain. *Nature* 429 883–891. 10.1038/nature02661 15190254

[B21] Lucin K. M. Wyss-Coray T. (2009). Immune activation in brain aging and neurodegeneration: too much or too little? *Neuron* 64 110–122. 10.1016/j.neuron.2009.08.039 19840553 PMC2834890

[B22] Lyu Y. Li Q. (2016). A semi-parametric statistical model for integrating gene expression profiles across different platforms. *BMC Bioinform*. 17:5. 10.1186/s12859-015-0847-y PMC4895261 26818110

[B23] McKinnon P. J. (2013). Maintaining genome stability in the nervous system. *Nat. Neurosci.* 16 1523–1529. 10.1038/nn.3537 24165679 PMC4112580

[B24] Montagne A. Barnes S. R. Sweeney M. D. Halliday M. R. Sagare A. P. Zhao Z. (2015). Blood-brain barrier breakdown in the aging human hippocampus. *Neuron* 85 296–302. 10.1016/j.neuron.2014.12.032 25611508 PMC4350773

[B25] Nagahara A. H. Merrill D. A. Coppola G. Tsukada S. Schroeder B. E. Shaked G. M. (2009). Neuroprotective effects of brain-derived neurotrophic factor in rodent and primate models of Alzheimer’s disease. *Nat. Med.* 15 331–337. 10.1038/nm.1912 19198615 PMC2838375

[B26] Roberts A. Trapnell C. Donaghey J. Rinn J. L. Pachter L. (2011). Improving RNA-Seq expression estimates by correcting for fragment bias. *Genom. Biol.* 12:R22. 10.1186/gb-2011-12-3-r22 21410973 PMC3129672

[B27] Santos R. X. Correia S. C. Zhu X. Smith M. A. Moreira P. I. Castellani R. J. (2013). Mitochondrial DNA oxidative damage and repair in aging and Alzheimer’s disease. *Antioxid. Redox Signal*. 18 2444–2457. 10.1089/ars.2012.5039 23216311 PMC3671662

[B28] Szklarczyk D. Gable A. L. Lyon D. Junge A. Wyder S. Huerta-Cepas J. (2019). STRING v11: protein–protein association networks with increased coverage, supporting functional discovery in genome-wide experimental datasets. *Nucleic Acids Res.* 47 D607–D613. 10.1093/nar/gky1131 30476243 PMC6323986

[B29] Toro-Domínguez D. Martorell-Marugán J. López-Domínguez R. García-Moreno A. González-Rumayor V. Alarcón-Riquelme M. E. (2019). ImaGEO: integrative gene expression meta-analysis from GEO database. *Bioinformatics* 35 880–882. 10.1093/bioinformatics/bty721 30137226

[B30] Van der Meer D. Rokicki J. Kaufmann T. Córdova-Palomera A. Moberget T. Alnæs D. (2020). Brain scans from 21,297 individuals reveal the genetic architecture of hippocampal subfield volumes. *Mol. Psychiatry* 25 3053–3065. 10.1038/s41380-018-0262-7 30279459 PMC6445783

[B31] Zhao Z. Ong L. K. Johnson S. Nilsson M. Walker F. R. (2017). Chronic stress induced disruption of the peri-infarct neurovascular unit following experimentally induced photothrombotic stroke. *J. Cereb. Blood Flow Metab.* 37 3709–3724. 10.1177/0271678X17696100 28304184 PMC5718325

