# Supplementary material for: Gene Expression Profiles of the Aging Rat Hippocampus Imply Altered Immunoglobulin Dynamics
Source: Front Neurosci. 2022 May 25;16:915907. doi: 10.3389/fnins.2022.915907 (PMC9174800; doi:10.3389/fnins.2022.915907)
Supplement: Supplementary file 1 [file Table_1.DOCX]

**Table S1.** Characteristics of the gene expression datasets included in the analysis.

| **Characteristics** | **Gene expression datasets** | | | | |
| --- | --- | --- | --- | --- | --- |
|  | **GSE14505** | **GSE20219** | **GSE14723** | **GSE14724** | **GSE14725** |
| Sex | Male | Male | Male | Male | Male |
| Strain model | WT | Fischer 344 | Long-Evans | Long-Evans | Long-Evans |
| Control samples  (*n*, age) | *n* = 4, 5-months  old | *n* = 8, 7-8 months  old | *n* = 9, 8-9 months  old | *n* = 9, 8-9 months  old | *n* = 8, 8-9 months  old |
| Test samples  (*n*, age) | *n* = 4, 24-months  old | *n* = 7, 21-22 months  old | *n* = 6, 24-26 months old | *n* = 6, 24-26 months old | *n* = 6, 24-26 months old |
| Hippocampal  samples | Whole hippocampus | Dorsal and ventral regions | CA1 region | CA3 region | Dentate gyrus |
| Experimental conditions | Vehicle infusion or sham surgery controls | Naïve controls and  tests | Naïve controls and tests | Naïve controls and tests | Naïve controls and tests |
| Microarray  platform | GPL1355 | GPL1355 | GPL1355 | GPL1355 | GPL1355 |

**Table S2.** Differentially expressed genes of the hippocampus between young (5-8 months) and aged (21-26 months) rats.

| **Gene ID** | ***P*-value** | ***Z*-score** | **Gene name** |
| --- | --- | --- | --- |
| *Upregulated* |  |  |  |
| A2M | 2.03E-05 | 5.36 | alpha-2-macroglobulin |
| AADAT | 5.06E-12 | 8.09 | aminoadipate aminotransferase |
| ACER2 | 8.66E-04 | 4.48 | alkaline ceramidase 2 |
| ACSL3 | 3.33E-06 | 5.75 | acyl-CoA synthetase long chain family member 3 |
| ADGRE1 | 6.64E-05 | 5.09 | adhesion G protein-coupled receptor E1 |
| ADPGK | 1.83E-02 | 3.52 | ADP dependent glucokinase |
| AEBP1 | 2.24E-04 | 4.81 | AE binding protein 1 |
| AFF1 | 4.00E-03 | 4.03 | AF4/FMR2 family member 1 |
| AGT | 5.28E-06 | 5.65 | angiotensinogen |
| AIF1 | 1.32E-02 | 3.64 | allograft inflammatory factor 1 |
| ALB | 2.24E-05 | 5.33 | albumin |
| ALDH16A1 | 6.81E-03 | 3.87 | aldehyde dehydrogenase 16 family member A1 |
| ANLN | 2.92E-05 | 5.27 | anillin actin binding protein |
| ANP32B | 2.44E-03 | 4.19 | acidic nuclear phosphoprotein 32 family member B |
| ANXA3 | 1.53E-06 | 5.90 | annexin A3 |
| ANXA4 | 8.35E-06 | 5.55 | annexin A4 |
| ANXA5 | 6.58E-06 | 5.61 | annexin A5 |
| APOD | 2.69E-02 | 3.37 | apolipoprotein D |
| ARHGEF10 | 1.66E-03 | 4.30 | Rho guanine nucleotide exchange factor 10 |
| ARRDC2 | 3.89E-02 | 3.23 | arrestin domain containing 2 |
| ATP6V0E1 | 4.32E-02 | 3.18 | ATPase H+ transporting V0 subunit e1 |
| B2M | 1.48E-04 | 4.90 | beta-2-microglobulin |
| B3GNT2 | 2.43E-02 | 3.42 | UDP-GlcNAc:betaGal beta-1,3-N-acetylglucosaminyltransferase 2 |
| BCAS1 | 3.11E-05 | 5.25 | brain enriched myelin associated protein 1 |
| BHLHE41 | 3.70E-06 | 5.73 | basic helix-loop-helix family member e41 |
| BLNK | 9.68E-05 | 5.00 | B cell linker |
| BMP6 | 4.47E-03 | 4.00 | bone morphogenetic protein 6 |
| C1QA | 3.47E-09 | 7.00 | complement C1q A chain |
| C1QB | 5.43E-04 | 4.60 | complement C1q B chain |
| C1QC | 2.90E-06 | 5.79 | complement C1q C chain |
| C3 | 4.27E-11 | 7.76 | complement C3 |
| C4A | 3.70E-07 | 6.17 | complement C4A (Rodgers blood group) |
| CAPG | 6.23E-05 | 5.10 | capping actin protein, gelsolin like |
| CAPN2 | 2.80E-04 | 4.76 | calpain 2 |
| CCDC120 | 3.81E-02 | 3.24 | coiled-coil domain containing 120 |
| CCDC81 | 1.98E-02 | 3.49 | coiled-coil domain containing 81 |
| CCN2 | 1.25E-05 | 5.46 | cellular communication network factor 2 |
| CD302 | 1.52E-09 | 7.15 | CD302 molecule |
| CD33 | 7.32E-03 | 3.84 | CD33 molecule |
| CD53 | 3.98E-07 | 6.15 | CD53 molecule |
| CD63 | 4.00E-03 | 4.03 | CD63 molecule |
| CD74 | 1.36E-08 | 6.75 | CD74 molecule |
| CD82 | 3.76E-02 | 3.24 | CD82 molecule |
| CD83 | 1.87E-03 | 4.26 | CD83 molecule |
| CDC40 | 4.67E-02 | 3.14 | cell division cycle 40 |
| CDH13 | 4.72E-02 | 3.13 | cadherin 13 |
| CDH23 | 2.31E-05 | 5.32 | cadherin related 23 |
| CDHR2 | 3.89E-02 | 3.23 | cadherin related family member 2 |
| CFH | 7.28E-03 | 3.84 | complement factor H |
| CHI3L1 | 4.78E-09 | 6.92 | chitinase 3 like 1 |
| CIB1 | 2.14E-02 | 3.46 | calcium and integrin binding 1 |
| CLDN10 | 5.31E-04 | 4.61 | claudin 10 |
| CLU | 3.15E-02 | 3.31 | clusterin |
| CMBL | 7.20E-05 | 5.07 | carboxymethylenebutenolidase homolog |
| CNDP1 | 1.50E-02 | 3.60 | carnosine dipeptidase 1 |
| COBL | 9.72E-03 | 3.76 | cordon-bleu WH2 repeat protein |
| CPEB1 | 6.98E-03 | 3.86 | cytoplasmic polyadenylation element binding protein 1 |
| CPPED1 | 1.60E-03 | 4.31 | calcineurin like phosphoesterase domain containing 1 |
| CPSF4 | 5.56E-03 | 3.94 | cleavage and polyadenylation specific factor 4 |
| CREBRF | 2.19E-02 | 3.46 | CREB3 regulatory factor |
| CRYAB | 1.26E-07 | 6.36 | crystallin alpha B |
| CSF1R | 4.80E-03 | 3.98 | colony stimulating factor 1 receptor |
| CSRP1 | 4.31E-03 | 4.01 | cysteine and glycine rich protein 1 |
| CSTB | 1.36E-03 | 4.36 | cystatin B |
| CTSA | 1.43E-03 | 4.34 | cathepsin A |
| CTSB | 1.13E-03 | 4.41 | cathepsin B |
| CTSD | 2.96E-05 | 5.26 | cathepsin D |
| CTSH | 1.14E-02 | 3.70 | cathepsin H |
| CTSS | 8.61E-09 | 6.83 | cathepsin S |
| CTSZ | 2.38E-08 | 6.65 | cathepsin Z |
| CYB5R2 | 6.80E-06 | 5.59 | cytochrome b5 reductase 2 |
| CYSTM1 | 1.64E-03 | 4.30 | cysteine rich transmembrane module containing 1 |
| DCTD | 1.25E-05 | 5.47 | dCMP deaminase |
| DDB2 | 4.41E-02 | 3.16 | damage specific DNA binding protein 2 |
| DDT | 3.50E-02 | 3.27 | D-dopachrome tautomerase |
| DECR1 | 1.52E-02 | 3.59 | 2,4-dienoyl-CoA reductase 1 |
| DHRS4 | 6.46E-03 | 3.88 | dehydrogenase/reductase 4 |
| EGLN3 | 2.54E-02 | 3.40 | egl-9 family hypoxia inducible factor 3 |
| ENPP4 | 7.93E-03 | 3.81 | ectonucleotide pyrophosphatase/phosphodiesterase 4 |
| EPDR1 | 1.25E-02 | 3.66 | ependymin related 1 |
| F3 | 7.20E-05 | 5.07 | coagulation factor III, tissue factor |
| FAH | 5.29E-03 | 3.95 | fumarylacetoacetate hydrolase |
| FAM177A1 | 2.77E-02 | 3.36 | family with sequence similarity 177 member A1 |
| FAM189A2 | 4.84E-03 | 3.98 | family with sequence similarity 189 member A2 |
| FCER1G | 4.80E-10 | 7.34 | Fc epsilon receptor Ig |
| FCGR2A | 2.85E-02 | 3.35 | Fc gamma receptor IIa |
| FCGR2B | 4.78E-09 | 6.93 | Fc gamma receptor IIb |
| FCGR3A | 3.36E-02 | 3.29 | Fc gamma receptor IIIa |
| FGL2 | 1.25E-05 | 5.46 | fibrinogen like 2 |
| FLCN | 6.69E-03 | 3.87 | folliculin |
| FN1 | 2.54E-02 | 3.40 | fibronectin 1 |
| FUCA1 | 2.13E-05 | 5.34 | alpha-L-fucosidase 1 |
| FXYD1 | 2.12E-05 | 5.35 | FXYD domain containing ion transport regulator 1 |
| FXYD3 | 3.10E-02 | 3.32 | FXYD domain containing ion transport regulator 3 |
| FXYD5 | 3.36E-02 | 3.29 | FXYD domain containing ion transport regulator 5 |
| GADD45B | 5.96E-03 | 3.91 | growth arrest and DNA damage inducible beta |
| GALM | 2.33E-02 | 3.43 | galactose mutarotase |
| GALNT6 | 6.26E-03 | 3.89 | polypeptide N-acetylgalactosaminyltransferase 6 |
| GCA | 3.55E-02 | 3.26 | grancalcin |
| GCSH | 3.15E-02 | 3.31 | glycine cleavage system protein H |
| GFAP | 2.10E-09 | 7.09 | glial fibrillary acidic protein |
| GIPR | 8.95E-03 | 3.78 | gastric inhibitory polypeptide receptor |
| GOLM1 | 1.55E-02 | 3.59 | golgi membrane protein 1 |
| GPATCH4 | 1.16E-02 | 3.70 | G-patch domain containing 4 (gene/pseudogene) |
| GPLD1 | 2.33E-02 | 3.43 | glycosylphosphatidylinositol specific phospholipase D1 |
| GPNMB | 4.80E-10 | 7.34 | glycoprotein nmb |
| GPR137B | 1.71E-02 | 3.55 | G protein-coupled receptor 137B |
| GPR84 | 1.87E-03 | 4.26 | G protein-coupled receptor 84 |
| GPRC5B | 7.92E-03 | 3.82 | G protein-coupled receptor class C group 5 member B |
| GRN | 3.34E-02 | 3.29 | granulin precursor |
| GRXCR1 | 3.04E-06 | 5.77 | glutaredoxin and cysteine rich domain containing 1 |
| GSTA1 | 7.49E-07 | 6.04 | glutathione S-transferase alpha 1 |
| GSTP1 | 8.23E-11 | 7.61 | glutathione S-transferase pi 1 |
| GUSB | 2.00E-02 | 3.49 | glucuronidase beta |
| HAPLN2 | 2.61E-02 | 3.39 | hyaluronan and proteoglycan link protein 2 |
| HEXA | 3.20E-03 | 4.11 | hexosaminidase subunit alpha |
| HEXB | 1.33E-03 | 4.36 | hexosaminidase subunit beta |
| HHATL | 4.59E-02 | 3.14 | hedgehog acyltransferase like |
| HPS1 | 4.99E-03 | 3.97 | HPS1 biogenesis of lysosomal organelles complex 3 subunit 1 |
| HSD17B11 | 7.31E-03 | 3.84 | hydroxysteroid 17-beta dehydrogenase 11 |
| HSPB1 | 9.33E-04 | 4.46 | heat shock protein family B (small) member 1 |
| HTRA1 | 4.07E-04 | 4.67 | HtrA serine peptidase 1 |
| ID4 | 2.11E-02 | 3.47 | inhibitor of DNA binding 4, HLH protein |
| IGF1R | 4.31E-02 | 3.18 | insulin like growth factor 1 receptor |
| IGSF1 | 3.23E-02 | 3.30 | immunoglobulin superfamily member 1 |
| IL18 | 2.79E-04 | 4.76 | interleukin 18 |
| IL33 | 1.66E-02 | 3.56 | interleukin 33 |
| IRF9 | 1.30E-02 | 3.65 | interferon regulatory factor 9 |
| ITGB2 | 2.48E-03 | 4.18 | integrin subunit beta 2 |
| ITGB5 | 3.79E-03 | 4.05 | integrin subunit beta 5 |
| ITIH3 | 1.84E-03 | 4.27 | inter-alpha-trypsin inhibitor heavy chain 3 |
| KCNJ16 | 8.63E-05 | 5.03 | potassium inwardly rectifying channel subfamily J member 16 |
| KLHDC7A | 2.96E-02 | 3.33 | kelch domain containing 7A |
| LAMP2 | 4.18E-04 | 4.66 | lysosomal associated membrane protein 2 |
| LAP3 | 7.44E-03 | 3.83 | leucine aminopeptidase 3 |
| LAPTM4B | 5.83E-03 | 3.92 | lysosomal protein transmembrane 4 beta |
| LAPTM5 | 3.70E-06 | 5.73 | lysosomal protein transmembrane 5 |
| LCAT | 6.00E-03 | 3.91 | lecithin-cholesterol acyltransferase |
| LGI4 | 4.11E-05 | 5.19 | leucine rich repeat LGI family member 4 |
| LILRA3 | 9.82E-03 | 3.75 | leukocyte immunoglobulin like receptor A3 |
| LMOD1 | 6.16E-03 | 3.90 | leiomodin 1 |
| MAOB | 5.74E-04 | 4.58 | monoamine oxidase B |
| MAP7 | 6.89E-04 | 4.53 | microtubule associated protein 7 |
| MARCHF2 | 2.19E-04 | 4.81 | membrane associated ring-CH-type finger 2 |
| MAST4 | 3.38E-02 | 3.28 | microtubule associated serine/threonine kinase family member 4 |
| MCM7 | 4.72E-02 | 3.13 | minichromosome maintenance complex component 7 |
| METTL7A | 1.23E-06 | 5.96 | methyltransferase like 7A |
| MGST1 | 2.02E-08 | 6.68 | microsomal glutathione S-transferase 1 |
| MICAL1 | 3.54E-03 | 4.08 | microtubule associated monooxygenase, calponin and LIM domain containing 1 |
| MKNK1 | 2.85E-02 | 3.35 | MAPK interacting serine/threonine kinase 1 |
| MLC1 | 1.50E-02 | 3.60 | modulator of VRAC current 1 |
| MOXD1 | 2.19E-04 | 4.82 | monooxygenase DBH like 1 |
| MPEG1 | 5.75E-04 | 4.58 | macrophage expressed 1 |
| MPHOSPH6 | 1.04E-02 | 3.73 | M-phase phosphoprotein 6 |
| MSN | 1.33E-02 | 3.64 | moesin |
| MT2A | 2.19E-02 | 3.46 | metallothionein 2A |
| MTMR10 | 4.24E-02 | 3.19 | myotubularin related protein 10 |
| MYH14 | 9.34E-04 | 4.46 | myosin heavy chain 14 |
| MYRF | 1.71E-02 | 3.55 | myelin regulatory factor |
| NCKAP1L | 1.25E-02 | 3.66 | NCK associated protein 1 like |
| NDRG1 | 4.37E-02 | 3.17 | N-myc downstream regulated 1 |
| NFE2L2 | 5.97E-04 | 4.57 | NFE2 like bZIP transcription factor 2 |
| NIBAN2 | 1.98E-02 | 3.50 | niban apoptosis regulator 2 |
| NPC2 | 5.06E-12 | 8.08 | NPC intracellular cholesterol transporter 2 |
| NPPA | 3.71E-03 | 4.06 | natriuretic peptide A |
| NPR2 | 1.98E-02 | 3.50 | natriuretic peptide receptor 2 |
| NT5E | 3.84E-02 | 3.23 | 5'-nucleotidase ecto |
| NUDT2 | 4.37E-02 | 3.17 | nudix hydrolase 2 |
| OAF | 6.26E-03 | 3.89 | out at first homolog |
| OTUD7B | 2.11E-02 | 3.47 | OTU deubiquitinase 7B |
| OXSR1 | 3.19E-04 | 4.73 | oxidative stress responsive kinase 1 |
| P2RY12 | 4.59E-02 | 3.14 | purinergic receptor P2Y12 |
| PACSIN3 | 4.31E-02 | 3.18 | protein kinase C and casein kinase substrate in neurons 3 |
| PADI2 | 4.82E-03 | 3.98 | peptidyl arginine deiminase 2 |
| PAIP2B | 3.62E-03 | 4.07 | poly(A) binding protein interacting protein 2B |
| PAQR6 | 6.82E-03 | 3.86 | progestin and adipoQ receptor family member 6 |
| PAQR7 | 3.50E-02 | 3.27 | progestin and adipoQ receptor family member 7 |
| PARP4 | 4.37E-02 | 3.17 | poly(ADP-ribose) polymerase family member 4 |
| PCDHB12 | 1.87E-03 | 4.26 | protocadherin beta 12 |
| PCDHB9 | 3.06E-03 | 4.12 | protocadherin beta 9 |
| PCSK6 | 7.84E-03 | 3.82 | proprotein convertase subtilisin/kexin type 6 |
| PDE8A | 3.18E-02 | 3.31 | phosphodiesterase 8A |
| PDK1 | 1.43E-03 | 4.34 | pyruvate dehydrogenase kinase 1 |
| PDLIM1 | 3.90E-02 | 3.22 | PDZ and LIM domain 1 |
| PDLIM4 | 1.59E-03 | 4.31 | PDZ and LIM domain 4 |
| PDRG1 | 1.43E-03 | 4.34 | p53 and DNA damage regulated 1 |
| PEPD | 2.63E-02 | 3.38 | peptidase D |
| PHLDB1 | 4.86E-02 | 3.11 | pleckstrin homology like domain family B member 1 |
| PHYHD1 | 3.46E-03 | 4.08 | phytanoyl-CoA dioxygenase domain containing 1 |
| PIR | 8.67E-03 | 3.79 | pirin |
| PLA2G4A | 2.02E-02 | 3.48 | phospholipase A2 group IVA |
| PLAAT3 | 4.84E-03 | 3.98 | phospholipase A and acyltransferase 3 |
| PLAG1 | 2.41E-02 | 3.42 | PLAG1 zinc finger |
| PLD4 | 1.60E-07 | 6.32 | phospholipase D family member 4 |
| PLEC | 1.72E-02 | 3.55 | plectin |
| PLEK | 2.67E-03 | 4.16 | pleckstrin |
| PLEKHB1 | 3.81E-07 | 6.16 | pleckstrin homology domain containing B1 |
| PLIN4 | 3.65E-03 | 4.06 | perilipin 4 |
| PLPP2 | 4.85E-02 | 3.12 | phospholipid phosphatase 2 |
| PLS1 | 1.62E-05 | 5.41 | plastin 1 |
| PMEL | 4.38E-06 | 5.69 | premelanosome protein |
| PMP22 | 1.16E-02 | 3.69 | peripheral myelin protein 22 |
| PON2 | 2.24E-05 | 5.33 | paraoxonase 2 |
| PPP1R1B | 6.16E-03 | 3.90 | protein phosphatase 1 regulatory inhibitor subunit 1B |
| PPT1 | 1.82E-03 | 4.27 | palmitoyl-protein thioesterase 1 |
| PRTFDC1 | 4.31E-02 | 3.18 | phosphoribosyl transferase domain containing 1 |
| PSMB8 | 6.04E-04 | 4.56 | proteasome 20S subunit beta 8 |
| PSME1 | 1.30E-03 | 4.37 | proteasome activator subunit 1 |
| PSME2 | 4.32E-02 | 3.18 | proteasome activator subunit 2 |
| PTP4A3 | 1.34E-05 | 5.45 | protein tyrosine phosphatase 4A3 |
| PTPDC1 | 3.89E-02 | 3.22 | protein tyrosine phosphatase domain containing 1 |
| PTPN11 | 5.69E-06 | 5.63 | protein tyrosine phosphatase non-receptor type 11 |
| PYCARD | 1.17E-02 | 3.69 | PYD and CARD domain containing |
| QDPR | 2.29E-07 | 6.26 | quinoid dihydropteridine reductase |
| RAPGEF3 | 2.72E-02 | 3.37 | Rap guanine nucleotide exchange factor 3 |
| RASGRP3 | 7.01E-06 | 5.58 | RAS guanyl releasing protein 3 |
| RASSF4 | 2.47E-05 | 5.31 | Ras association domain family member 4 |
| RCAN2 | 5.72E-03 | 3.93 | regulator of calcineurin 2 |
| RENBP | 9.46E-04 | 4.45 | renin binding protein |
| RIDA | 2.62E-03 | 4.17 | reactive intermediate imine deaminase A homolog |
| RIN2 | 1.25E-02 | 3.66 | Ras and Rab interactor 2 |
| RLBP1 | 9.29E-05 | 5.01 | retinaldehyde binding protein 1 |
| RNASET2 | 3.25E-08 | 6.59 | ribonuclease T2 |
| S100A13 | 3.00E-03 | 4.12 | S100 calcium binding protein A13 |
| S100A4 | 2.35E-03 | 4.20 | S100 calcium binding protein A4 |
| S100B | 3.71E-03 | 4.06 | S100 calcium binding protein B |
| SALL1 | 3.18E-05 | 5.24 | spalt like transcription factor 1 |
| SCN4B | 1.98E-02 | 3.49 | sodium voltage-gated channel beta subunit 4 |
| SCPEP1 | 1.32E-02 | 3.64 | serine carboxypeptidase 1 |
| SERPING1 | 1.82E-03 | 4.27 | serpin family G member 1 |
| SGK1 | 2.97E-02 | 3.33 | serum/glucocorticoid regulated kinase 1 |
| SGK3 | 4.13E-02 | 3.20 | serum/glucocorticoid regulated kinase family member 3 |
| SHC4 | 9.05E-05 | 5.02 | SHC adaptor protein 4 |
| SIGLEC5 | 1.24E-02 | 3.67 | sialic acid binding Ig like lectin 5 |
| SKAP2 | 2.42E-06 | 5.82 | src kinase associated phosphoprotein 2 |
| SLC15A2 | 1.25E-02 | 3.66 | solute carrier family 15 member 2 |
| SLC25A13 | 1.17E-03 | 4.40 | solute carrier family 25 member 13 |
| SLC3A2 | 2.51E-02 | 3.41 | solute carrier family 3 member 2 |
| SLC66A3 | 7.44E-03 | 3.83 | solute carrier family 66 member 3 |
| SMAD3 | 2.63E-02 | 3.38 | SMAD family member 3 |
| SMOC1 | 2.23E-02 | 3.45 | SPARC related modular calcium binding 1 |
| SNAPC2 | 3.55E-03 | 4.07 | small nuclear RNA activating complex polypeptide 2 |
| SNAPIN | 3.50E-02 | 3.27 | SNAP associated protein |
| SNX5 | 4.48E-02 | 3.15 | sorting nexin 5 |
| SORT1 | 3.34E-02 | 3.29 | sortilin 1 |
| SPOCK3 | 2.49E-03 | 4.18 | SPARC (osteonectin), cwcv and kazal like domains proteoglycan 3 |
| SQOR | 5.60E-03 | 3.94 | sulfide quinone oxidoreductase |
| SREBF1 | 7.93E-03 | 3.81 | sterol regulatory element binding transcription factor 1 |
| SRPRB | 8.86E-03 | 3.78 | SRP receptor subunit beta |
| ST3GAL6 | 2.33E-02 | 3.43 | ST3 beta-galactoside alpha-2,3-sialyltransferase 6 |
| STAT2 | 3.74E-02 | 3.25 | signal transducer and activator of transcription 2 |
| STXBP3 | 7.32E-03 | 3.84 | syntaxin binding protein 3 |
| SUN2 | 3.33E-04 | 4.72 | Sad1 and UNC84 domain containing 2 |
| TAF13 | 1.14E-02 | 3.70 | TATA-box binding protein associated factor 13 |
| TGFA | 1.25E-02 | 3.66 | transforming growth factor alpha |
| TJP2 | 1.41E-02 | 3.62 | tight junction protein 2 |
| TMBIM1 | 3.09E-02 | 3.32 | transmembrane BAX inhibitor motif containing 1 |
| TMED10 | 1.79E-02 | 3.53 | transmembrane p24 trafficking protein 10 |
| TMEM176A | 2.86E-05 | 5.27 | transmembrane protein 176A |
| TMEM176B | 2.15E-06 | 5.84 | transmembrane protein 176B |
| TMEM255A | 1.25E-02 | 3.66 | transmembrane protein 255A |
| TMEM63A | 4.37E-02 | 3.17 | transmembrane protein 63A |
| TMOD1 | 1.16E-02 | 3.69 | tropomodulin 1 |
| TNNC2 | 4.18E-04 | 4.66 | troponin C2, fast skeletal type |
| TNXB | 2.58E-03 | 4.17 | tenascin XB |
| TP53BP2 | 6.77E-06 | 5.60 | tumor protein p53 binding protein 2 |
| TREM2 | 7.34E-04 | 4.52 | triggering receptor expressed on myeloid cells 2 |
| TRIM34 | 1.76E-02 | 3.54 | tripartite motif containing 34 |
| TSC22D4 | 1.87E-03 | 4.26 | TSC22 domain family member 4 |
| TSPAN12 | 1.81E-02 | 3.53 | tetraspanin 12 |
| TSPO | 4.96E-05 | 5.15 | translocator protein |
| TYROBP | 9.92E-09 | 6.80 | transmembrane immune signaling adaptor TYROBP |
| UBR7 | 1.97E-02 | 3.50 | ubiquitin protein ligase E3 component n-recognin 7 |
| USP54 | 7.73E-06 | 5.56 | ubiquitin specific peptidase 54 |
| VKORC1 | 1.12E-03 | 4.41 | vitamin K epoxide reductase complex subunit 1 |
| VWC2 | 3.84E-02 | 3.23 | von Willebrand factor C domain containing 2 |
| VWF | 1.98E-02 | 3.49 | von Willebrand factor |
| WIPI1 | 3.96E-02 | 3.22 | WD repeat domain, phosphoinositide interacting 1 |
|  |  |  |  |
| *Downregulated* |  |  |  |
| ABCA3 | 2.54E-02 | -3.40 | ATP binding cassette subfamily A member 3 |
| ACO1 | 2.18E-03 | -4.22 | aconitase 1 |
| ACSS2 | 2.60E-05 | -5.29 | acyl-CoA synthetase short chain family member 2 |
| ACTR1A | 2.11E-02 | -3.47 | actin related protein 1A |
| ACVR2A | 4.10E-03 | -4.03 | activin A receptor type 2A |
| ADAM11 | 4.72E-02 | -3.13 | ADAM metallopeptidase domain 11 |
| ADCY5 | 1.19E-02 | -3.68 | adenylate cyclase 5 |
| AGRN | 4.43E-04 | -4.65 | agrin |
| AGXT2 | 4.32E-02 | -3.18 | alanine--glyoxylate aminotransferase 2 |
| ALDH1B1 | 1.66E-02 | -3.56 | aldehyde dehydrogenase 1 family member B1 |
| AP2M1 | 2.67E-03 | -4.16 | adaptor related protein complex 2 subunit mu 1 |
| AP2S1 | 4.32E-02 | -3.18 | adaptor related protein complex 2 subunit sigma 1 |
| APCDD1 | 1.92E-02 | -3.51 | APC down-regulated 1 |
| ARF5 | 1.21E-02 | -3.68 | ADP ribosylation factor 5 |
| ARHGDIG | 5.43E-04 | -4.60 | Rho GDP dissociation inhibitor gamma |
| ATP6V0E2 | 2.85E-02 | -3.35 | ATPase H+ transporting V0 subunit e2 |
| B3GAT2 | 4.83E-04 | -4.63 | beta-1,3-glucuronyltransferase 2 |
| B4GAT1 | 1.22E-02 | -3.67 | beta-1,4-glucuronyltransferase 1 |
| BCAN | 4.79E-02 | -3.12 | brevican |
| BCL11B | 3.36E-02 | -3.29 | BAF chromatin remodeling complex subunit BCL11B |
| BHLHE22 | 2.67E-02 | -3.38 | basic helix-loop-helix family member e22 |
| BLMH | 3.36E-02 | -3.29 | bleomycin hydrolase |
| BOK | 1.45E-02 | -3.61 | BCL2 family apoptosis regulator BOK |
| BTBD10 | 1.33E-02 | -3.64 | BTB domain containing 10 |
| CACNA1A | 2.33E-02 | -3.43 | calcium voltage-gated channel subunit alpha1 A |
| CACNA2D3 | 1.62E-03 | -4.31 | calcium voltage-gated channel auxiliary subunit alpha2delta 3 |
| CALML4 | 7.44E-03 | -3.83 | calmodulin like 4 |
| CAMKV | 4.59E-02 | -3.14 | CaM kinase like vesicle associated |
| CARS1 | 3.76E-02 | -3.24 | cysteinyl-tRNA synthetase 1 |
| CD200 | 5.50E-04 | -4.59 | CD200 molecule |
| CDC42 | 2.84E-02 | -3.35 | cell division cycle 42 |
| CDIPT | 6.98E-03 | -3.86 | CDP-diacylglycerol--inositol 3-phosphatidyltransferase |
| CHD3 | 2.93E-02 | -3.34 | chromodomain helicase DNA binding protein 3 |
| CHN2 | 4.67E-02 | -3.14 | chimerin 2 |
| CLDN11 | 1.30E-02 | -3.65 | claudin 11 |
| CNRIP1 | 6.26E-03 | -3.89 | cannabinoid receptor interacting protein 1 |
| COL4A1 | 1.72E-02 | -3.54 | collagen type IV alpha 1 chain |
| COL4A5 | 5.52E-06 | -5.64 | collagen type IV alpha 5 chain |
| COL5A3 | 4.55E-02 | -3.15 | collagen type V alpha 3 chain |
| CRLF1 | 8.81E-04 | -4.47 | cytokine receptor like factor 1 |
| CRMP1 | 1.63E-02 | -3.57 | collapsin response mediator protein 1 |
| CRYM | 3.89E-02 | -3.23 | crystallin mu |
| CSRP2 | 2.03E-05 | -5.36 | cysteine and glycine rich protein 2 |
| CTNNA2 | 1.16E-02 | -3.69 | catenin alpha 2 |
| CTNNB1 | 9.80E-03 | -3.75 | catenin beta 1 |
| CYP51A1 | 2.65E-03 | -4.16 | cytochrome P450 family 51 subfamily A member 1 |
| CYYR1 | 4.33E-02 | -3.18 | cysteine and tyrosine rich 1 |
| DAB2 | 4.35E-03 | -4.01 | DAB adaptor protein 2 |
| DCAF7 | 2.22E-02 | -3.45 | DDB1 and CUL4 associated factor 7 |
| DCN | 3.07E-02 | -3.32 | decorin |
| DDHD1 | 2.42E-02 | -3.42 | DDHD domain containing 1 |
| DLL1 | 4.37E-02 | -3.17 | delta like canonical Notch ligand 1 |
| DNAJA1 | 4.13E-02 | -3.20 | DnaJ heat shock protein family (Hsp40) member A1 |
| DNAJB11 | 3.84E-02 | -3.23 | DnaJ heat shock protein family (Hsp40) member B11 |
| DPP3 | 2.69E-02 | -3.37 | dipeptidyl peptidase 3 |
| DSTN | 3.79E-02 | -3.24 | destrin, actin depolymerizing factor |
| DUSP18 | 4.72E-02 | -3.13 | dual specificity phosphatase 18 |
| DYNLL1 | 4.37E-02 | -3.17 | dynein light chain LC8-type 1 |
| DYNLT1 | 2.86E-03 | -4.14 | dynein light chain Tctex-type 1 |
| EHBP1 | 1.16E-02 | -3.70 | EH domain binding protein 1 |
| EIF4A1 | 1.78E-02 | -3.53 | eukaryotic translation initiation factor 4A1 |
| ELP1 | 3.98E-02 | -3.21 | elongator acetyltransferase complex subunit 1 |
| ELP6 | 1.22E-02 | -3.67 | elongator acetyltransferase complex subunit 6 |
| EMC6 | 2.69E-03 | -4.15 | ER membrane protein complex subunit 6 |
| EMID1 | 3.95E-02 | -3.22 | EMI domain containing 1 |
| ENO1 | 3.89E-02 | -3.23 | enolase 1 |
| EPHA3 | 3.84E-02 | -3.23 | EPH receptor A3 |
| EPHA5 | 4.22E-02 | -3.19 | EPH receptor A5 |
| EPRS1 | 4.48E-02 | -3.15 | glutamyl-prolyl-tRNA synthetase 1 |
| F2R | 1.85E-03 | -4.27 | coagulation factor II thrombin receptor |
| FABP5 | 2.96E-02 | -3.33 | fatty acid binding protein 5 |
| FAT1 | 1.04E-02 | -3.73 | FAT atypical cadherin 1 |
| FBLN2 | 5.75E-04 | -4.58 | fibulin 2 |
| FBXW9 | 1.36E-02 | -3.63 | F-box and WD repeat domain containing 9 |
| FEZ1 | 3.93E-02 | -3.22 | fasciculation and elongation protein zeta 1 |
| FGF13 | 2.48E-03 | -4.19 | fibroblast growth factor 13 |
| FGF9 | 6.12E-03 | -3.90 | fibroblast growth factor 9 |
| FGFBP3 | 3.10E-02 | -3.32 | fibroblast growth factor binding protein 3 |
| FSTL5 | 5.11E-06 | -5.66 | follistatin like 5 |
| FUBP1 | 4.04E-02 | -3.21 | far upstream element binding protein 1 |
| FXYD7 | 1.69E-02 | -3.55 | FXYD domain containing ion transport regulator 7 |
| FYN | 2.42E-02 | -3.42 | FYN proto-oncogene, Src family tyrosine kinase |
| GALNT11 | 4.37E-02 | -3.17 | polypeptide N-acetylgalactosaminyltransferase 11 |
| GAMT | 1.47E-02 | -3.60 | guanidinoacetate N-methyltransferase |
| GAP43 | 1.30E-02 | -3.65 | growth associated protein 43 |
| GDF10 | 4.32E-02 | -3.18 | growth differentiation factor 10 |
| GLRA2 | 8.69E-08 | -6.43 | glycine receptor alpha 2 |
| GLRX3 | 2.54E-02 | -3.40 | glutaredoxin 3 |
| GLRX5 | 1.21E-02 | -3.68 | glutaredoxin 5 |
| GNG2 | 3.52E-02 | -3.26 | G protein subunit gamma 2 |
| GNG3 | 1.84E-02 | -3.52 | G protein subunit gamma 3 |
| GP1BB | 6.00E-03 | -3.91 | glycoprotein Ib platelet subunit beta |
| GPR162 | 1.25E-02 | -3.67 | G protein-coupled receptor 162 |
| GSPT2 | 3.94E-02 | -3.22 | G1 to S phase transition 2 |
| H3-3B | 1.09E-02 | -3.72 | H3.3 histone B |
| HDAC6 | 3.98E-02 | -3.21 | histone deacetylase 6 |
| HES5 | 3.37E-02 | -3.28 | hes family bHLH transcription factor 5 |
| HPRT1 | 4.37E-02 | -3.17 | hypoxanthine phosphoribosyltransferase 1 |
| HPS3 | 4.40E-02 | -3.16 | HPS3 biogenesis of lysosomal organelles complex 2 subunit 1 |
| HSBP1 | 6.55E-03 | -3.88 | heat shock factor binding protein 1 |
| IGF2 | 4.61E-05 | -5.17 | insulin like growth factor 2 |
| ILDR2 | 4.59E-02 | -3.14 | immunoglobulin like domain containing receptor 2 |
| ISCA1 | 2.83E-02 | -3.35 | iron-sulfur cluster assembly 1 |
| ITGA11 | 4.37E-02 | -3.17 | integrin subunit alpha 11 |
| KANSL2 | 1.27E-02 | -3.65 | KAT8 regulatory NSL complex subunit 2 |
| KCNMB4 | 3.76E-02 | -3.24 | potassium calcium-activated channel subfamily M regulatory beta subunit 4 |
| KDELR3 | 4.79E-02 | -3.12 | KDEL endoplasmic reticulum protein retention receptor 3 |
| KDM2B | 2.79E-02 | -3.36 | lysine demethylase 2B |
| KIF2A | 4.86E-02 | -3.11 | kinesin family member 2A |
| KLHL5 | 4.72E-02 | -3.13 | kelch like family member 5 |
| LDHA | 1.16E-02 | -3.69 | lactate dehydrogenase A |
| LIX1 | 5.79E-03 | -3.92 | limb and CNS expressed 1 |
| LMO2 | 3.50E-02 | -3.27 | LIM domain only 2 |
| LMO4 | 4.31E-02 | -3.18 | LIM domain only 4 |
| LPL | 3.19E-05 | -5.24 | lipoprotein lipase |
| LRRC59 | 2.77E-02 | -3.36 | leucine rich repeat containing 59 |
| LRRTM1 | 2.14E-03 | -4.23 | leucine rich repeat transmembrane neuronal 1 |
| LXN | 1.66E-02 | -3.56 | latexin |
| MAGED1 | 3.49E-02 | -3.27 | MAGE family member D1 |
| MAL | 4.32E-02 | -3.18 | mal, T cell differentiation protein |
| MARCHF3 | 4.32E-02 | -3.18 | membrane associated ring-CH-type finger 3 |
| MEST | 2.13E-02 | -3.47 | mesoderm specific transcript |
| MLLT11 | 1.50E-03 | -4.33 | MLLT11 transcription factor 7 cofactor |
| MMP14 | 2.22E-02 | -3.45 | matrix metallopeptidase 14 |
| MPC2 | 6.96E-03 | -3.86 | mitochondrial pyruvate carrier 2 |
| MPZL1 | 8.32E-05 | -5.04 | myelin protein zero like 1 |
| MTPAP | 1.12E-02 | -3.71 | mitochondrial poly(A) polymerase |
| MYADM | 1.60E-02 | -3.57 | myeloid associated differentiation marker |
| MYCN | 4.72E-02 | -3.13 | MYCN proto-oncogene, bHLH transcription factor |
| MYL6 | 3.36E-03 | -4.09 | myosin light chain 6 |
| MYO16 | 1.27E-03 | -4.38 | myosin XVI |
| MYO5B | 2.61E-02 | -3.39 | myosin VB |
| MZT2B | 1.87E-04 | -4.85 | mitotic spindle organizing protein 2B |
| NALF2 | 2.57E-03 | -4.17 | NALCN channel auxiliary factor 2 |
| NDN | 9.02E-08 | -6.42 | necdin, MAGE family member |
| NDUFA9 | 1.36E-02 | -3.63 | NADH:ubiquinone oxidoreductase subunit A9 |
| NEDD8 | 4.55E-02 | -3.15 | NEDD8 ubiquitin like modifier |
| NNAT | 1.22E-04 | -4.95 | neuronatin |
| NOLC1 | 2.61E-02 | -3.38 | nucleolar and coiled-body phosphoprotein 1 |
| NREP | 3.70E-07 | -6.17 | neuronal regeneration related protein |
| OXCT1 | 2.65E-02 | -3.38 | 3-oxoacid CoA-transferase 1 |
| PALMD | 6.74E-03 | -3.87 | palmdelphin |
| PDHX | 9.90E-03 | -3.75 | pyruvate dehydrogenase complex component X |
| PHOSPHO1 | 2.58E-02 | -3.39 | phosphoethanolamine/phosphocholine phosphatase 1 |
| PIGX | 3.52E-02 | -3.26 | phosphatidylinositol glycan anchor biosynthesis class X |
| PLA2G12A | 4.37E-02 | -3.17 | phospholipase A2 group XIIA |
| PNLIP | 4.73E-02 | -3.13 | pancreatic lipase |
| POLR2G | 3.07E-02 | -3.32 | RNA polymerase II subunit G |
| PPP1R10 | 3.45E-02 | -3.28 | protein phosphatase 1 regulatory subunit 10 |
| PPP2R2B | 9.43E-04 | -4.45 | protein phosphatase 2 regulatory subunit Bbeta |
| PRDM8 | 7.31E-03 | -3.84 | PR/SET domain 8 |
| PREP | 1.87E-03 | -4.26 | prolyl endopeptidase |
| PROM1 | 9.90E-03 | -3.75 | prominin 1 |
| PSMD8 | 2.54E-02 | -3.40 | proteasome 26S subunit, non-ATPase 8 |
| PTPRB | 2.10E-02 | -3.47 | protein tyrosine phosphatase receptor type B |
| PTPRO | 3.47E-09 | -6.99 | protein tyrosine phosphatase receptor type O |
| RAB6B | 2.97E-03 | -4.13 | RAB6B, member RAS oncogene family |
| RALYL | 1.77E-03 | -4.28 | RALY RNA binding protein like |
| RAP1GAP | 5.79E-03 | -3.92 | RAP1 GTPase activating protein |
| REEP1 | 5.88E-04 | -4.57 | receptor accessory protein 1 |
| REXO2 | 2.60E-02 | -3.39 | RNA exonuclease 2 |
| RGS14 | 1.71E-02 | -3.55 | regulator of G protein signaling 14 |
| RGS5 | 4.79E-02 | -3.12 | regulator of G protein signaling 5 |
| RHOT1 | 1.15E-02 | -3.70 | ras homolog family member T1 |
| RNF128 | 3.95E-02 | -3.22 | ring finger protein 128 |
| RNF145 | 2.71E-02 | -3.37 | ring finger protein 145 |
| RNF180 | 4.04E-04 | -4.68 | ring finger protein 180 |
| RNF41 | 2.22E-02 | -3.45 | ring finger protein 41 |
| RNF6 | 1.33E-02 | -3.63 | ring finger protein 6 |
| RNPEP | 8.67E-03 | -3.79 | arginyl aminopeptidase |
| RRAGA | 2.75E-03 | -4.15 | Ras related GTP binding A |
| RRAS2 | 5.95E-03 | -3.91 | RAS related 2 |
| RTN1 | 4.50E-02 | -3.15 | reticulon 1 |
| RYBP | 2.30E-02 | -3.44 | RING1 and YY1 binding protein |
| SCAMP1 | 1.60E-03 | -4.31 | secretory carrier membrane protein 1 |
| SEMA3C | 5.48E-11 | -7.69 | semaphorin 3C |
| SERF1A | 4.43E-02 | -3.16 | small EDRK-rich factor 1A |
| SEZ6 | 2.00E-02 | -3.49 | seizure related 6 homolog |
| SFXN1 | 3.00E-02 | -3.33 | sideroflexin 1 |
| SFXN3 | 3.73E-03 | -4.06 | sideroflexin 3 |
| SLC15A4 | 9.58E-05 | -5.00 | solute carrier family 15 member 4 |
| SLC17A7 | 1.90E-02 | -3.51 | solute carrier family 17 member 7 |
| SLC25A33 | 2.64E-03 | -4.16 | solute carrier family 25 member 33 |
| SLC2A3 | 1.41E-02 | -3.62 | solute carrier family 2 member 3 |
| SLC31A1 | 3.46E-03 | -4.08 | solute carrier family 31 member 1 |
| SLC35D3 | 4.30E-02 | -3.19 | solute carrier family 35 member D3 |
| SLC39A10 | 1.27E-04 | -4.93 | solute carrier family 39 member 10 |
| SLC49A4 | 8.95E-03 | -3.78 | solute carrier family 49 member 4 |
| SLC6A1 | 4.14E-02 | -3.20 | solute carrier family 6 member 1 |
| SLC7A1 | 1.02E-02 | -3.74 | solute carrier family 7 member 1 |
| SLC9A3R2 | 9.58E-05 | -5.00 | SLC9A3 regulator 2 |
| SLCO1A2 | 4.37E-02 | -3.17 | solute carrier organic anion transporter family member 1A2 |
| SLCO1C1 | 6.26E-08 | -6.49 | solute carrier organic anion transporter family member 1C1 |
| SMAP1 | 9.32E-03 | -3.77 | small ArfGAP 1 |
| SMARCE1 | 1.53E-05 | -5.42 | SWI/SNF related, matrix associated, actin dependent regulator of chromatin, subfamily e, member 1 |
| SNCA | 8.53E-06 | -5.54 | synuclein alpha |
| SORCS2 | 2.96E-02 | -3.33 | sortilin related VPS10 domain containing receptor 2 |
| SOX17 | 2.84E-02 | -3.35 | SRY-box transcription factor 17 |
| SRA1 | 2.23E-02 | -3.45 | steroid receptor RNA activator 1 |
| ST8SIA3 | 2.70E-02 | -3.37 | ST8 alpha-N-acetyl-neuraminide alpha-2,8-sialyltransferase 3 |
| STIP1 | 9.82E-03 | -3.75 | stress induced phosphoprotein 1 |
| STMN2 | 8.76E-05 | -5.03 | stathmin 2 |
| STRADB | 4.75E-02 | -3.13 | STE20 related adaptor beta |
| STRN | 2.63E-02 | -3.38 | striatin |
| STX1A | 3.94E-02 | -3.22 | syntaxin 1A |
| SUCLA2 | 4.37E-02 | -3.17 | succinate-CoA ligase ADP-forming subunit beta |
| SULT4A1 | 4.37E-02 | -3.17 | sulfotransferase family 4A member 1 |
| SYMPK | 1.30E-02 | -3.65 | symplekin scaffold protein |
| SYN1 | 5.25E-03 | -3.96 | synapsin I |
| TCFL5 | 1.88E-02 | -3.51 | transcription factor like 5 |
| TDG | 4.37E-02 | -3.17 | thymine DNA glycosylase |
| TEK | 1.98E-02 | -3.49 | TEK receptor tyrosine kinase |
| TFRC | 3.66E-05 | -5.21 | transferrin receptor |
| TM7SF2 | 1.66E-02 | -3.56 | transmembrane 7 superfamily member 2 |
| TMED4 | 3.89E-02 | -3.22 | transmembrane p24 trafficking protein 4 |
| TMEM163 | 7.73E-04 | -4.50 | transmembrane protein 163 |
| TMEM38A | 4.31E-02 | -3.18 | transmembrane protein 38A |
| TMUB2 | 2.93E-02 | -3.34 | transmembrane and ubiquitin like domain containing 2 |
| TPGS1 | 4.86E-02 | -3.11 | tubulin polyglutamylase complex subunit 1 |
| TPP2 | 2.60E-02 | -3.39 | tripeptidyl peptidase 2 |
| TRAF3 | 4.31E-02 | -3.18 | TNF receptor associated factor 3 |
| TRIB2 | 1.27E-04 | -4.93 | tribbles pseudokinase 2 |
| TRIL | 5.73E-03 | -3.93 | TLR4 interactor with leucine rich repeats |
| TRIM27 | 3.73E-02 | -3.25 | tripartite motif containing 27 |
| TST | 1.72E-02 | -3.54 | thiosulfate sulfurtransferase |
| TTC9B | 1.22E-04 | -4.95 | tetratricopeptide repeat domain 9B |
| TTYH3 | 4.46E-02 | -3.16 | tweety family member 3 |
| TUBA4A | 1.68E-02 | -3.56 | tubulin alpha 4a |
| TUBB2A | 3.83E-02 | -3.24 | tubulin beta 2A class IIa |
| TUBB2B | 1.77E-05 | -5.39 | tubulin beta 2B class IIb |
| TUBB3 | 2.54E-02 | -3.40 | tubulin beta 3 class III |
| TUBB4B | 1.64E-02 | -3.57 | tubulin beta 4B class IVb |
| TXLNA | 2.77E-02 | -3.36 | taxilin alpha |
| TXNDC16 | 1.52E-02 | -3.59 | thioredoxin domain containing 16 |
| UBA1 | 4.63E-02 | -3.14 | ubiquitin like modifier activating enzyme 1 |
| UBL4A | 1.11E-03 | -4.41 | ubiquitin like 4A |
| UGT8 | 4.79E-02 | -3.12 | UDP glycosyltransferase 8 |
| USO1 | 2.36E-02 | -3.43 | USO1 vesicle transport factor |
| USP7 | 3.37E-02 | -3.28 | ubiquitin specific peptidase 7 |
| VANGL2 | 2.05E-02 | -3.48 | VANGL planar cell polarity protein 2 |
| VTN | 5.57E-03 | -3.94 | vitronectin |
| XPO7 | 3.36E-02 | -3.29 | exportin 7 |
| YKT6 | 5.79E-03 | -3.92 | YKT6 v-SNARE homolog |
| ZC3H8 | 4.32E-02 | -3.18 | zinc finger CCCH-type containing 8 |
| ZDBF2 | 1.52E-02 | -3.59 | zinc finger DBF-type containing 2 |
| ZDHHC16 | 4.72E-02 | -3.13 | zinc finger DHHC-type palmitoyltransferase 16 |
| ZFAND3 | 4.85E-02 | -3.12 | zinc finger AN1-type containing 3 |
| ZNRF1 | 2.00E-02 | -3.49 | zinc and ring finger 1 |
| ZYG11B | 2.73E-02 | -3.37 | zyg-11 family member B, cell cycle regulator |

**Table S3.** Clustering gene modules in the protein-protein interaction network of differentially expressed genes of the hippocampus between young (5-8 months) and aged (21-26 months) rats.

| **Cluster** | **MCODE Score** | **Gene density** | **Gene edges** |
| --- | --- | --- | --- |
| 1 | 16.667 | 19 | 150 |
| 2 | 5.867 | 16 | 44 |
| 3 | 5.111 | 19 | 46 |
| 4 | 5 | 5 | 10 |
| 5 | 4 | 4 | 6 |
| 6 | 3.5 | 13 | 21 |
| 7 | 3.333 | 4 | 5 |
| 8 | 3 | 3 | 3 |
| 9 | 3 | 3 | 3 |
| 10 | 3 | 7 | 9 |
| 11 | 3 | 7 | 9 |
| 12 | 3 | 3 | 3 |
| 13 | 3 | 3 | 3 |
| 14 | 2.824 | 18 | 24 |
| 15 | 2.8 | 6 | 7 |
| 16 | 2.545 | 12 | 14 |

**Table S4.** Characteristics of the highest-ranked clustering gene module in the protein-protein interaction network of differentially expressed genes of the hippocampus between young (5-8 months) and aged (21-26 months) rats.

| **Gene ID** | ***P*-value** | ***Z*-score** | **Gene name** |
| --- | --- | --- | --- |
| AIF1 | 1.32E-02 | 3.64 | allograft inflammatory factor 1 |
| C1QA | 3.47E-09 | 7.00 | complement C1q A chain |
| C1QB | 5.43E-04 | 4.60 | complement C1q B chain |
| C1QC | 2.90E-06 | 5.79 | complement C1q C chain |
| CD53 | 3.98E-07 | 6.15 | CD53 molecule |
| CD74 | 1.36E-08 | 6.75 | CD74 molecule |
| CSF1R | 4.79E-03 | 3.98 | colony stimulating factor 1 receptor |
| CTSS | 8.61E-09 | 6.83 | cathepsin S |
| FCER1G | 4.80E-10 | 7.34 | Fc epsilon receptor Ig |
| FCGR2A | 2.85E-02 | 3.35 | Fc gamma receptor IIa |
| FCGR2B | 4.78E-09 | 6.93 | Fc gamma receptor IIb |
| FCGR3A | 3.36E-02 | 3.29 | Fc gamma receptor IIIa |
| FGL2 | 1.25E-05 | 5.46 | fibrinogen like 2 |
| ITGB2 | 2.48E-03 | 4.18 | integrin subunit beta 2 |
| LAPTM5 | 3.70E-06 | 5.73 | lysosomal protein transmembrane 5 |
| MPEG1 | 5.75E-04 | 4.58 | macrophage expressed 1 |
| PLEK | 2.67E-03 | 4.16 | pleckstrin |
| TREM2 | 7.34E-04 | 4.52 | triggering receptor expressed on myeloid cells 2 |
| TYROBP | 9.92E-09 | 6.80 | transmembrane immune signaling adaptor TYROBP |

**Table S5.** Functional enrichment of the highest-ranked clustering gene module in the protein-protein interaction network of differentially expressed genes of the hippocampus between young (5-8 months) and aged (21-26 months) rats. The top 10 gene ontology annotations based on biological process, molecular function, and cellular component, are shown.

| **GO Term** | **Description** | **Ontology** | **Genes** | ***P*-value** |
| --- | --- | --- | --- | --- |
| Immune response | Any immune system process that functions in the calibrated response of an organism to a potential internal or invasive threat. | BP | 17 | 1.71E-17 |
| Regulation of immune system process | Any process that modulates the frequency, rate, or extent of an immune system process. | BP | 16 | 1.71E-17 |
| Regulation of immune response | Any process that modulates the frequency, rate or extent of the immune response, the immunological reaction of an organism to an immunogenic stimulus. | BP | 14 | 1.28E-16 |
| Positive regulation of immune system process | Any process that activates or increases the frequency, rate, or extent of an immune system process. | BP | 13 | 1.33E-14 |
| Defence response | Reactions, triggered in response to the presence of a foreign body or the occurrence of an injury, which result in restriction of damage to the organism attacked or prevention/recovery from the infection caused by the attack. | BP | 15 | 1.33E-14 |
| Immune effector process | Any process of the immune system that executes a component of an immune response. An effector immune process takes place after its activation. | BP | 12 | 1.96E-14 |
| Defence response to other organism | Reactions triggered in response to the presence of another organism that act to protect the cell or organism from damage caused by that organism. | BP | 13 | 1.15E-13 |
| Innate immune response | Innate immune responses are defense responses mediated by germline encoded components that directly recognize components of potential pathogens. | BP | 12 | 3.56E-13 |
| Regulation of cell activation | Any process that modulates the frequency, rate or extent of cell activation, the change in the morphology or behavior of a cell resulting from exposure to an activating factor such as a cellular or soluble ligand. | BP | 11 | 1.32E-12 |
| Leukocyte mediated immunity | Any process involved in the carrying out of an immune response by a leukocyte. | BP | 10 | 1.6E-12 |
| IgG binding | Binding to an immunoglobulin of an IgG isotype. | MF | 4 | 1.92E-08 |
| Immunoglobulin binding | Binding to an immunoglobulin. | MF | 4 | 2.30E-07 |
| Amyloid beta binding | Binding to an amyloid-beta peptide/protein. | MF | 5 | 2.30E-07 |
| Protein containing complex binding | Binding to a macromolecular complex. | MF | 9 | 8.27E-07 |
| Immunoglobulin receptor activity | Combining with the Fc region of an immunoglobulin protein and transmitting the signal from one side of the membrane to the other to initiate a change in cell activity. | MF | 3 | 1.04E-06 |
| Molecular transducer activity | A compound molecular function in which an effector function is controlled by one or more regulatory components. | MF | 8 | 5.77E-05 |
| Peptide binding | Binding to a peptide, an organic compound comprising two or more amino acids linked by peptide bonds. | MF | 5 | 8.46E-05 |
| Immune receptor activity | Receiving a signal and transmitting it in a cell to initiate an immune response. | MF | 4 | 1.34E-04 |
| Amide binding | Binding to an amide, any derivative of an oxoacid in which an acidic hydroxy group has been replaced by an amino or substituted amino group. | MF | 5 | 1.98E-04 |
| Identical protein binding | Binding to an identical protein or proteins. | MF | 6 | 3.51E-02 |
| Intrinsic component of plasma membrane | The component of the plasma membrane consisting of the gene products and protein complexes having either part of their peptide sequence embedded in the hydrophobic region of the membrane or some other covalently attached group such as a GPI anchor that is similarly embedded in the membrane. | CC | 10 | 1.25E-06 |
| Cell surface | The external part of the cell wall and/or plasma membrane. | CC | 8 | 2.19E-06 |
| Secretory granule | A small subcellular vesicle, surrounded by a membrane, that is formed from the Golgi apparatus and contains a highly concentrated protein destined for secretion. Secretory granules move towards the periphery of the cell and upon stimulation, their membranes fuse with the cell membrane, and their protein load is exteriorized. Processing of the contained protein may take place in secretory granules. | CC | 7 | 2.53E-05 |
| Secretory vesicle | A cytoplasmic, membrane bound vesicle that is capable of fusing to the plasma membrane to release its contents into the extracellular space. | CC | 7 | 5.44E-05 |
| Secretory granule membrane | The lipid bilayer surrounding a secretory granule. | CC | 5 | 5.44E-05 |
| Vesicle membrane | The lipid bilayer surrounding any membrane-bounded vesicle in the cell. | CC | 7 | 1.09E-04 |
| Tertiary granule | A secretory granule that contains cathepsin and gelatinase and is readily exocytosed upon cell activation; found primarily in mature neutrophil cells. | CC | 4 | 1.34E-04 |
| Collagen containing extracellular matrix | An extracellular matrix consisting mainly of proteins (especially collagen) and glycosaminoglycans (mostly as proteoglycans) that provides not only essential physical scaffolding for the cellular constituents but can also initiate crucial biochemical and biomechanical cues required for tissue morphogenesis, differentiation and homeostasis. The components are secreted by cells in the vicinity and form a sheet underlying or overlying cells such as endothelial and epithelial cells. | CC | 5 | 1.59E-04 |
| Ficolin 1 rich granule | Highly exocytosable gelatinase-poor granules found in neutrophils and rich in ficolin-1. Ficolin-1 is released from neutrophil granules by stimulation with fMLP or PMA, and the majority becomes associated with the surface membrane of the cells and can be detected by flow cytometry. | CC | 4 | 1.61E-04 |
| External side of plasma membrane | The leaflet of the plasma membrane that faces away from the cytoplasm and any proteins embedded or anchored in it or attached to its surface. | CC | 5 | 1.61E-04 |
